# Time Perception of an Artwork’s Manipulation Is Distorted by Patients With Parkinson’s Disease

**DOI:** 10.3389/fnint.2019.00006

**Published:** 2019-03-08

**Authors:** Márcia Regina Motta, Vitor Tumas, José Lino Oliveira Bueno

**Affiliations:** ^1^Department of Psychology, Psychobiology Division, Faculty of Philosophy, Sciences and Letters of Ribeirão Preto, University of São Paulo, Ribeirão Preto, Brazil; ^2^Department of Neuroscience and Behavior Sciences, Movement Disorder Division, School of Medicine of Ribeirão Preto, University of São Paulo, Ribeirão Preto, Brazil

**Keywords:** timing, parkinsonism, exploratory behavior, motor activity, touch, art

## Abstract

**Objectives:** In artwork appreciation situations, individuals often show altered time perception. We tested the hypothesis that Parkinson’s disease (PD) patients present movement patterns that have an impact on the time perception of artwork manipulation time. We predicted that, compared to healthy controls (non-PD), differences in the exploratory behavior of patients would evoke alteration of artwork manipulation time perception.

**Methods:** Ten PD patients and 10 non-PD participants manipulated two reproductions of artwork with different complexity levels from the series “Bichos” by Lygia Clark. Subsequently, participants performed a verbal estimation regarding the temporal duration of their manipulations. The exploratory behavior was analyzed.

**Results:** All participants overestimated the artwork manipulation time. However, PD patients, regardless of the artwork’s level of complexity, showed shorter manipulation time and minor time overestimation compared to the non-PD participants. PD patients touched the artworks more often, especially the more complex artworks, than the non-PD participants; in contrast, PD patients moved the artworks less often, particularly the less complex artwork.

**Conclusion:** PD patients showed an altered perception of artwork manipulation time. This suggests that exploratory behavior influenced temporal estimation. Besides, it is likely that PD patients had presented a decreased ability to manage attention during the task, which interfered in the cognitive reconstruction of its duration. Considered altogether, these appointments indicate that, as a result of cognitive and motor deficits, PD patients showed impairment in temporal information processing. The exploratory behavior facilitated the understanding of these results and processes in terms of motor-timing operations of the basal ganglia-thalamocortical system.

## Introduction

Parkinson’s disease (PD) is a movement disorder characterized by present motor alterations such as tremor at rest, rigidity, bradykinesia, and postural instability; notwithstanding, non-motor manifestation may be present, such as autonomic nervous system disorder, cognitive, psychiatric, and sleep alteration, and sensory symptoms (Sveinbjornsdottir, 2016; Schapira et al., 2017; Tarakad and Jankovic, 2017). The neurobiology of PD is characterized by the atrophy of the substantia nigra and a depletion of dopamine-releasing neurons that project to the caudate-putamen. These structures have been considered to be closely related to temporal processing (Allman and Meck, 2012; Marinho et al., 2017). In fact, investigations on the neural mechanisms of temporal processing point to convergent results of studies in animals and humans that indicate temporal perception is dependent on the activation of basal ganglia and its thalamus-cortical path dependence on dopamine (Matell and Meck, 2004; Bakhurin et al., 2017; Emmons et al., 2017). This neural network is also involved in mechanisms associated with the voluntary action (Harrington and Jahanshahi, 2016).

The studies of PD patients’ perception of time not only show different dysfunctions in the temporal processing (Jones et al., 2008; Merchant et al., 2008; Wearden et al., 2008; Koch et al., 2009; Harrington et al., 2011) but also demonstrate that there is no consensus on whether the verbal temporal estimation is affected by the motor activity of PD patients (Pastor et al., 1992; Riesen and Schnider, 2001). Pastor et al. (1992) found that PD patients underestimated the intervals in time estimation tasks. In contrast, Riesen and Schnider (2001) found that PD patients estimated the time duration of a motor task with the same precision as the controls. Moreover, results from Torta et al. (2010), which involved motor activity, indicated that the ability to reproduce motor acts in PD patients can be dissociated from that of the reproduction of time intervals and can be improved by the administration of treatment. It is likely that some of the contradictory findings reported in the literature on PD patients’ perception of time” may be explained by the motor action required (Jones et al., 2008).

The present study is oriented by the New Experimental Aesthetics, which consists of the study of the artworks through empirical scientific methods in order to evaluate its effects on behavior (Berlyne, 1958, 1963, 1974). In this way, it is possible to study the effects of some attributes of artworks such as complexity level on esthetic experience. According to Berlyne (1958, 1963), features such as the amount of material or variety in a piece and irregularity of shape or arrangement can be covered by the term “complexity.” In the present study the complexity level of the artwork was related to the number of parts or pieces that the artwork has. The artwork considered to be more complex has a greater number of pieces than the artwork considered to be less complex. Several studies conducted in healthy participants have revealed that the appreciation of artworks influences the perception of time (Cocenas-Silva et al., 2011; Ramos and Bueno, 2012; Firmino and Bueno, 2016). In the visual arts, Nather and Bueno (2006, 2011, 2012b) showed that the suggestion of motion in a static image extends the subjective temporal experience.

The present study has three aims. The first is to investigate the effect of an artwork manipulation on the time perception in PD patients. In particular, it was investigated whether differences in temporal judgments in patients with PD compared to healthy controls are related to a motor task. Previous studies conducted with PD patients have already pointed out the influence of motor activity on time perception in the temporal reproduction task (Pastor et al., 1992; Malapani et al., 1998). Considering that PD patients present a disorder of structures related to temporal processing, i.e., basal ganglia dysfunction due to dopaminergic depletion, and consequent motor impairment, we predict that PD patients would present distortions in their temporal judgments due to impairment of their motor skills.

The second aim is to investigate the effect of the artwork manipulation with different levels of complexity on the time perception of PD patients. According to previous studies with healthy individuals, the degree of complexity of the artwork influences the time perception of its appreciation (Bueno et al., 2002; Firmino et al., 2009; Nather and Bueno, 2011; Nather et al., 2011; Firmino and Bueno, 2016). Moreover, studies involving the motor action and PD showed that PD patients present failure in motor information processing, which may be linked to attentional and memory deficits (Broeder et al., 2014; Gruszka et al., 2017; Nieuwhof et al., 2017; Sakurada et al., 2018). This evidence allowed us to predict impairment in the judgment of artworks’ manipulation time with different levels of complexity in PD patients. This may be related to the deficit in information processing, which is fundamental to distinguishing differences between artworks and estimating differences in the time spent manipulating the artwork.

The third aim is to investigate whether the manipulation of artwork by PD patients and healthy participants would present qualitative differences. Based on the analysis of movement categories, we predicted that the PD patients would have less use of categories of movements that would compromise the exploration of the artwork, whereas healthy participants would use more categories of movements that would allow them to better explore the artworks.

## Materials and Methods

### Participants

Ten patients with Kindly confirm whether the heading levels have been correctly identified.PD (7 males, 3 females) and 10 healthy subjects (4 males, 6 females) took part in this study. The Research Ethics Committee of the University of São Paulo approved the study (CAAE n° 68684917.6.0000.5407), and each participant signed an informed written consent upon inclusion in the study. Patients with a diagnosis of PD were recruited from the Ribeirão Preto School of Medicine Hospital, São Paulo, Brazil. A neurologist, specialist in movement disorders, made the diagnosis of PD according to the UK Brain Bank Criteria (Hughes et al., 1992). All patients were evaluated using the “Movement Disorders Society-Unified Parkinson’s disease Rating Scale” – (MDS-UPDRS), which indicates the severity of PD, and all patients were tested while they were on antiparkinsonian medication action (Goetz, 2010). Non-PD participants were recruited from the local community (Ribeirão Preto, São Paulo, Brazil; Table 1). These subjects were matched to PD patients on the basis of age (±2 years compared to the PD sample) but not on education. Exclusion criteria included possible dementia or cognitive impairment, significant history or current psychiatric disorders, historic of neurological disease or any condition (e.g., depression) that would interfere with testing (see Table 1).

**Table 1:** Demographic and clinical characteristics (mean and SD) are reported for the participants with Parkinson’s disease (PD) and non-Parkinson’s disease participants (N-PD).

	DP *n* = 10	N-PD *n* = 10
Age	57.3 (5.33)	54.4 (8.37)
Education	5.9 (3.90)	10.6 (2.22)ˆ*
UPDRS-MDS motor	29.6 (13.78)	–
MMSE	25.2 (2.66)	26.2 (1.62)
GDS	3.1 (1.85)	2.4 (1.96)

*UPDRS-MDS motor, Unified Parkinson’s disease Rating Scale-part III (maximum = 132); MMSE, Mini Mental State Examination (maximum = 30); and GDS, Geriatric Depression Scale (maximum = 15); ^∗^p = 0.005.*

The global cognitive functioning of all subjects was evaluated with the Mini-Mental State Examination (MMSE; Folstein et al., 1975) using appropriate cut off scores for suspected dementia (Brucki et al., 2003). The 15-item Geriatric Depression Scale (GDS; Yesavage et al., 1983) was used to diagnose depression in patients and healthy participants (Tumas et al., 2008).

All participants did not report any formal and systematic prior training in artistic activity.

The two groups (PD and non-PD) did not differ regarding age, MMSE and GDS (all *p’s* > 0.005). Differences were observed between PD patients and non-PD participants on education (*r* = −0.570, *p* = 0.005; Table 1).

### Equipment and Material

The PD patients and healthy participants were tested at the hospital in Ribeirão Preto, São Paulo, Brazil. During the task, participants were seated in front of a rotating bulkhead containing three compartments. In each compartment, different artwork stimuli were placed. A Panasonic HDC-HS80 camcorder was attached to a rod 55 cm above the manipulation site. E-prime^®^ 2.0 was used to run the experiment. The time spent by participants in manipulating the artworks was recorded by them using a key connected to a PC. The participants were tested during an experimental session that lasted approximately 40 min. The participants’ psychological information was collected by an assistant psychologist before the experiment.

Two reproductions of artworks from the series “Bichos” by Lygia Clark (Butler and Pérez-Oramas, 2014) were used as experimental stimuli. The artworks were composed of flat pieces of 2-mm-thick frosted aluminum. Each piece had the geometric shape of a scalene triangle, with dimensions 26 cm × 17 cm × 14.5 cm. The flat pieces were functionally related to each other, which allowed them to be moved in many configurations.

The less complex (LC) artwork had six flat pieces, and the more complex (MC) artwork had 10 flat pieces. According to the collative properties of stimuli (see Berlyne, 1963), LC artwork represents a lower level of complexity than the MC artwork. This attribution of level of complexity to these two stimuli was confirmed by participant judges of a previous study (Motta, 2016).

The training stimulus was composed of one flat piece of 2-mm-thick frosted aluminum, with the geometric shape of a scalene triangle, with dimensions 26 cm × 17 cm × 14.5 cm, bent at a 90° angle height.

### Manipulation Task and Verbal Estimation Task

The experiment begins with the training phase in which participants manipulated the training stimulus. The participants repeated the training as many times as they wanted before moving on to the experimental phase. The instructions for the training and the experimental phases were the same. The experimental phase consisted of the participants manipulating one stimulus at a time as long as they wanted. The presentation sequence of LC and MC artworks was randomly distributed to each participant. Each participant had only one trial for the manipulation of less complex artwork and more complex artwork. In order to record the duration of manipulation, participants were requested to press a key connected to the PC at the beginning and at the end of the manipulation of each artwork. After the task was completed with the manipulation of both stimuli, the participants were asked to make a verbal estimate of the duration of each artwork’s manipulation. Thus, the participants were tested under the retrospective paradigm, i.e., they received no prior notice about the estimation of the duration of manipulation to be made after the task (Grondin, 2010). The duration of the manipulation estimates was annotated by the experimenter. The manipulation of the stimuli was recorded for later analysis of the movements. These movements were analyzed according to categories of manipulation (described below).

### Categories of Manipulation

The exploratory activity of the participants was analyzed considering categories of manipulation. These categories were based on the recording of the manipulations. These were transcribed, and subsequently analyzed. Then, four categories were identified: Touch, Move, Release the piece of art (stimulus), and Change the artwork’s placement. The criterion used was based on the grasp-type classification of Lee and Jung (2014), which investigated the types of voluntary grasp commonly used according to the object, shape, size, and direction; thus, the movement of the hands and fingers was considered as performed by participants at the moment of grasping and manipulating the artwork. The definition of each category included the following: *Touch:* touch performed with the palm and/or fingers; *Move:* movement of the plates performed with the palm and fingers; *Release the piece of art:* when the artwork was dropped without any contact with hands; and *Change the artwork placement:* when the artwork was displaced from a local to another in the rotating bulkhead base.

### Data Analysis

#### Verbal Temporal Estimation of Manipulation Time

Since there is variation in the total time spent on the manipulation of artworks by participants, special methodologies were taken as the standardization of verbal temporal estimation using the following formula (Nather and Bueno, 2012a):

$$\text{WTEC}={\text{t}}_{\left(\text{r}\right)}-{\text{t}}_{\left(\text{e}\right)}/{\text{t}}_{\left(\text{t}\right)}$$

in which,

t(r) is the time spent by the participant for the manipulation,

t(e) is the verbal temporal estimation of the manipulation by the participant.

WTEC is the weighted temporal estimation coefficient, whose negative values were considered an overestimation and the positive values an underestimation.

#### Categories of Stimulus Manipulation

The filming of the four categories of the stimulus manipulation [Touch, Move, Release the piece of art (stimulus), and Change the artwork’s placement] was analyzed using the EthoLog 2.2 software, a tool that assists in the observation of behavior, registering its sequence, frequency, and duration (Ottoni, 2000).

Considering the variation in the manipulation time of artworks by participants, standardization was made to analyze the categories of manipulation using the following formula:

$${\text{P}}_{\left(\text{cat}\right)}={\text{t}}_{\left(\text{cat}\right)}/{\text{t}}_{\text{r}}{\;\mathrm{and FR}}_{\left(\text{cat}\right)}={\text{F}}_{\text{abs}}/{\text{t}}_{\text{r}}$$

in which,

P_(*cat*)_ is the proportion of each category,

t_*r*_ is the time spent by the participant to manipulate the artworks,

FR_(*cat*)_is the relative frequency of each category,

and F_*abs*_ is the absolute frequency of each category.

The categories of manipulation were described as absolute frequency, relative frequency, and proportion.

As the Shapiro-Wilk test did not show normal distribution of the data, we used non-parametric statistics, for comparisons of WTEC, time of manipulation and categories of manipulation of the stimuli. The Mann-Whitney *U* test was used for comparisons between the PD patients and non-PD participants for each stimulus (less complex and more complex stimulus). The Wilcoxon test was used for comparisons between less complex stimuli versus more complex for each group (PD and non-PD). All the results were presented in the Figures as averages to facilitate the description of the data, but the statistically significant differences were calculated by the non-parametric tests using the ranking. A *p*-value of 0.05 was considered statistically significant. It was calculated also the Effect size (r). *P*-values were not adjusted for multiple pairwise comparisons. This approach can potentially mask important findings. Also, the Effect size along with exact *p*-values allows readers to assess the significance (and statistical significance) of the results (Nakagawa, 2004; Armstrong, 2014). The SPSS statistical package for Windows (version 23.0, SPSS, Inc., Chicago, IL, United States) was used for the statistical analyses.

## Results

### Manipulation Time

**FIGURE 1:**
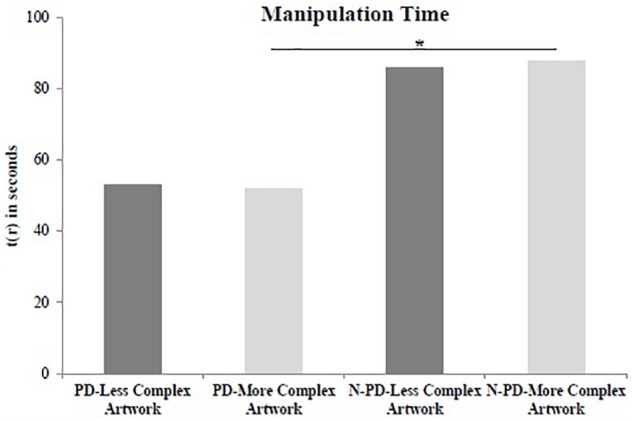
Time spent on manipulation. Time spent, in seconds [t_(*r*)_], on manipulation of the less complex artwork and more complex artwork by the participants with Parkinson’s disease (PD) and non-Parkinson’s disease (N-PD); ^∗^*p* < 0.005. The figure shows average data only to facilitate the observation of the differences, but the non-parametric statistical analysis considered the ranking of each value.

We compared PD patients and non-PD participants performance carrying out two different comparisons: less complex stimulus and more complex stimulus. There was a significant effect of group to the time spent to manipulate the more complex artwork (*U* = 18,000, *p* = 0.015, *r* = 0.321), but not for less complex artwork (*U* = 26,000, *p* = 0.075, *r* = 0.491), although in this case the Effect size has been moderate to relatively large. The non-PD group presented a greater time to manipulate the more complex artwork than PD group (PD = 52 s; non-PD = 88 s; Figure 1).

Comparisons within groups revealed no effect of the complexity of the artworks on manipulation time (*W* = 30,000, *p* = 0.799, *r* = 0.588) for PD participants (Figure 1), although both measures had a large Effect size. The non-PD participants did not show any difference for the manipulation time between the less complex artwork and more complex artwork (*W* = 31,000, *p* = 0.721, *r* = 0.891; Figure 1).

### Time Estimation (Values of WTEC)

**FIGURE 2:**
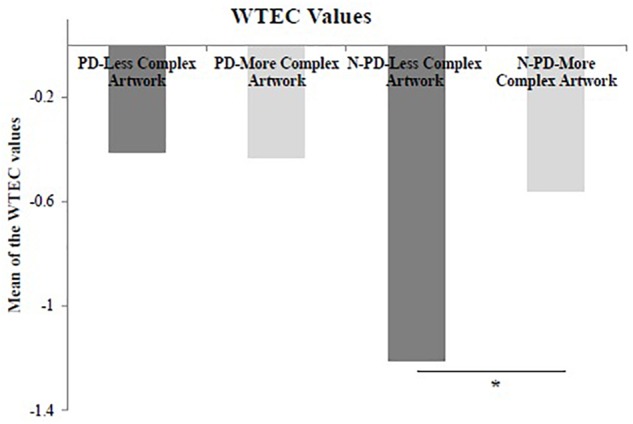
Mean of the weighted temporal estimation coefficient (WTEC) of participants with Parkinson’s disease (PD) and non-Parkinson’s disease (N-PD) related to temporal estimation of the manipulation of the less complex artwork and the more complex artwork. WTEC was computed as the ratio between the time spent by the participant for the manipulation discounting the verbal temporal estimation of the manipulation by the participant and the time spent by the participant for the manipulation. Negative values were considered to be overestimation, and the positive values were considered to be underestimation; ^∗^*p* < 0.005. The figure shows the averages only to facilitate the observation of the differences, but the non-parametric statistical analysis considered the classification of the ranking.

For the values of WTEC, both groups of participants showed negative value (DP = −0.42; non-PD = −0.89) to estimation of the manipulation time of the artworks (less complex and more complex). The results indicated that all participants overestimated the time spent manipulating artworks. Although, the participants with PD appeared to have estimated the shortest manipulation time compared to the control group, there was no statistical significance between the groups for the less complex stimulus (*U* = 66,000, *p* = 0.247, *r* = 0.139) nor for the more complex stimulus (*U* = 60,000, *p* = 0.481, *r* = 0.164; Figure 2).

Comparisons within groups revealed no effect of the complexity of the artworks on WTEC (*W* = 25,000, *p* = 0.799, *r* = 0.842) for PD participants (Figure 2), although both measures had a large Effect size. However, there was effect of complexity of the stimuli on the values of WTEC (*W* = 47,000, *p* = 0.047, *r* = 0.782) for the non-PD group. The participants of the non-PD group judged the manipulation time of the less complex artwork to be larger than the manipulation time of the more complex artwork (LC = −1.21; MC = −0.56; Figure 2).

### Categories of Manipulation

**FIGURE 3:**
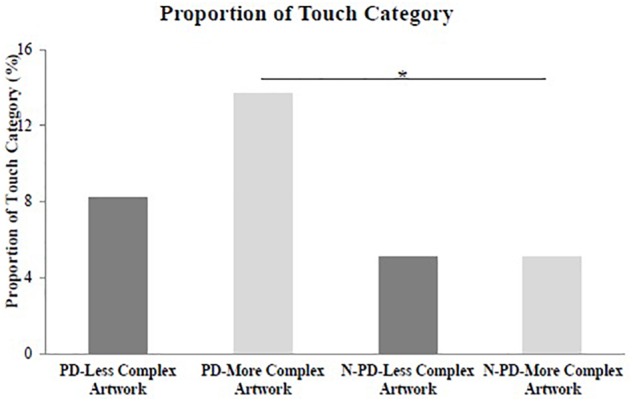
Proportion of Touch category. Proportion of touches in the less complex artwork and more complex artwork performed by the participants with Parkinson’s disease (PD) and non-Parkinson’s disease participants (N-PD). Proportion of Touch category was computed as the ratio between the time spent touching the artworks and the total time spent by the participant for the manipulation; ^∗^*p* < 0.005.

**FIGURE 4:**
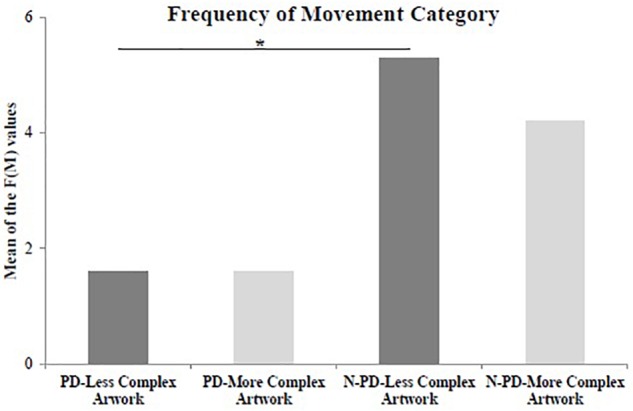
Mean of the frequency of Movement category [F_(M)_]. Mean of how many times the participants with Parkinson’s disease (PD) and non-Parkinson’s disease (N-PD) moved the less complex artwork and more complex artwork; ^∗^*p* < 0.005.

For standardized measures (absolute frequency, relative frequency, and proportion) to each category of manipulation (Touch, Move, Release the piece of art, and Change the artwork’s placement), there was a group effect for the proportion of the Touch category for the more complex artwork (*U* = 74,000, *p* = 0.017, *r* = 0.183). In addition, there was an effect for the frequency of the Movement category for the less complex artwork (*U* = 20,500, *p* = 0.043, *r* = 0.104). These findings indicate that PD patients presented a higher proportion of touches for the more complex artwork than non-PD participants (PD = 13.7%, non-PD = 5.1%). The PD patients moved the less complex artwork less often than the non-PD patients (PD = 1.66; non-PD = 5.30; Figure 3, 4). Although the Effect size was small for both the categories of movement.

Comparisons within groups revealed no effect of the complexity of the artworks on standardized measures to each category of manipulation for PD patients and for non-PD participants.

Differences in education and in the MMSE and GDS test scores may be related to the results found in the temporal estimation of manipulation time. The *t*-test for the Spearman correlation coefficient showed a moderate negative association between the education level and the estimate of the duration of manipulation of the less complex artwork by the PD group (*r* = −0.570, *p* = 0.005).

## Discussion

The estimation of the manipulation duration of the artworks was longer than the real duration (overestimation) for all participants. Our results are in accordance with studies that point out that the complexity (Block, 1978; Block et al., 2010; Sasayama, 2016), the cognitive requirement (McClain, 1983; Simchy-Gross and Margulis, 2018; Xu and David, 2018), and the amount of informational content (Hicks et al., 1976) of a task influence the time perception. Block (1978) found that a complex sequence of visual patterns was remembered to have a longer duration compared to a simple sequence. McClain (1983), using stimuli lists with different quantities of words that would be processed in tasks requiring different levels of complexity, found that the time estimation on the retrospective paradigm would be a direct function of the amount of information presented, regardless of the degree of complexity of its processing; then, McClain observed that when the words were presented more, the estimated duration was longer. Such data point out that the perception of the time judged increases in line with the increased cognitive requirement of an event that occurred in a time interval. On the other hand, Hicks et al. (1976) verified that the retrospective temporal judgment would not occur as a function of the amount of processed information. The authors argued that the judgment of time may be related to the amount of content recovered and highlighted that the poor content of information may be a factor that leads to the lack of effect on the duration remembered. The results of these studies lead us to infer that the overestimation of the manipulation time found in our research would be related to the complexity of the motor task and the complexity of the artworks, the cognitive requirement of attention and memory, and the amount of informational content to be processed.

The PD patients presented shorter overestimation than the non-PD participants. Although there was no difference between groups for the estimation of time, it should be noted that, the PD patients estimated time intervals more accurately than the controls. However, it is important to take into account the general context in which the temporal judgment occurred (Merchant et al., 2013; Merchant and Yarrow, 2016). Henceforth, some aspects must be considered.

The first aspect refers to significant differences found between the manipulation time of the artworks by PD and non-PD participants; the shorter duration of the artwork manipulation was presented by PD patients, whereas the longer duration of artwork manipulation was observed among non-PD participants. The time to perform the task may be related to motor aspects observed among PD patients. Torta et al. (2010) found that PD patients performed the motor task during a larger time than healthy individuals. This difference probably occurred due to the characteristic of the motor task: the task consisted of unscrewing a stud nut over the whole length. However, in comparison to their study, the motor demand was more difficult in our experiment: participants were requested to manipulate artworks with different levels of complexity. Although intra PD group comparisons did not show significant statistical differences between less complex artwork and more complex artwork, inter-group comparisons showed that PD patients presented shorter duration of manipulation of the more complex artwork than the non-PD participants. This suggests that the difficulty of the task may have been greater for the PD patients. Gentilucci and Negrotti (1999b) showed that the high demand for a motor task is reflected in the difficulty of executing a motor plane by patients with PD, who present motor program decay over time. Additionally, to support our interpretation of the task’s difficulty level, there was a significant difference for the manipulation time of the artwork with a more complex level: the PD patients presented a shorter duration of manipulation of the more complex artwork than the non-PD participants.

The second aspect is related to factors of time estimation that are not linked to the motor task (Gibbon et al., 1997; Malapani et al., 2002; Berry et al., 2014; Marchetti, 2014; Matthews and Meck, 2016). Thus, considerations about the contribution of the attentional process for time estimation are particularly important given that attention deficits have being demonstrated in PD, especially when patients have to rely on internal cues and strategies for performing the task (Brown and Marsden, 1990). In our study, the complexity of the motor task may have required a high level of attention, and this may have influenced time perception. This inference is supported by Riesen and Schnider (2001), who considered that attention is not affected by an automatic motor task such as a tapping task. They observed that PD patients estimated time intervals between 12 and 48 s as accurately as the controls. During these intervals, participants were requested to tap and, at the same time, read a random number on the computer screen at a rate of 1 Hz. Considering that tapping is a simpler motor task than a visuomotor task (such as the one used in our study), it is possible that PD patients have presented decreased skills in managing attention. That may indicate a distinction between automatic/passive and effort-demanding/active processes (Brown and Marsden, 1990), which points to important implications of attentional factors to time perception.

Besides that, some researchers point out the effect of a task and its processing on remembered duration (Hicks et al., 1976; McClain, 1983; Block and Zakay, 1997; Cohn-Sheehy and Ranganath, 2017; Teki et al., 2017) and consider that, in the retrospective timing, the judgment is based on a cognitive reconstruction of the task duration (Block, 1978). Prior to these studies, Ornstein (1969) proposed the theory of storage, suggesting that a complex task requires larger cognitive demand resulting in larger “storage size” in the memory, which would lengthen the remembered duration. Based on this, although our research does not directly address this aspect, the overestimation of the task time presented by participants may be related to the storage and retrieval of information linked to temporal memory processes (Malapani et al., 2002).

The third aspect that must be pointed out is that our patients’ performance might have been more accurate because they received their usual medication, which included L-dopa (precursor to the neurotransmitter dopamine). It is known that dopamine has been involved in temporal processing. It would act as a modulator in the cortico-striatal circuit influencing the processing of temporal information (Allman and Meck, 2012; Agostino and Cheng, 2016). Malapani et al. (1998), compared PD patients with healthy young and aged controls, showed that patients with PD who demonstrated deficits in accuracy and precision in temporal reproduction tasks, when under the administration of L-dopa had its deficits attenuated. Torta et al. (2010) compared PD patients under both L-dopa and non-L-dopa medication and found that the PD patients under the effect of medication demonstrated improve motor deficits in a motor task and in the temporal estimation skills. The authors showed the relation between motor activity, treatment, and time perception. Also, Pastor et al. (1992) compared PD patients with and without medication, observed a significant improvement in the PD patients’ performance under the effect of medication in a verbal estimation task. Thus, considering that the dopamine, as a modulator, tends to accelerate the time perception (Magalhães et al., 2018), reducing the overestimation, the L-dopa, in the present study, would have contributed to the shorter overestimation demonstrated by PD patients when compared with the non-PD participants, without, however, entirely restoring all cognitive performance, such as memory and attention, and motor performance. It should be borne in mind that the artwork manipulation produced temporal overestimation for both PD and non-PD patients, and the effect of the L-dopa was to affect the temporal processing of PD patients reducing the overestimation. The second aim of the present study was to investigate the effect of the manipulation of artworks with different levels of complexity on the time perception of PD patients. The results showed that the non-PD participants presented differences in the time estimation with the artworks: the time interval of the manipulation of the less complex artwork was perceived to be longer than the time interval of the manipulation of the more complex artwork. Studies with healthy individuals (Hicks et al., 1976; McClain, 1983; Block and Zakay, 1997; Sasayama, 2016; Simchy-Gross and Margulis, 2018; Xu and David, 2018) indicate that when the complexity of a task or stimulus is at a higher level, the cognitive requirement and the content of information to be processed are greater, which influences the time judgment: the time will be perceived as longer than the duration of the task. However, the manipulation of artworks with different levels of complexity did not result in differences in time perception of PD patients: the time intervals of the manipulation of the less complex artwork and the more complex artwork were perceived to have the same duration. This result may be related to the deficit in perceptual-motor tasks (Stern et al., 1983) and an impairment in the ability to process the information during the selection and execution of movements (Sanes, 1985). Sanes (1985) evaluated the capacity to process information during movement selection and execution in Parkinsonian patients and healthy participants in a task involving the movement of a hand-held stylus between two targets whose size and separation could be systematically varied. Results showed that PD patients cannot process equivalent amounts of information per unit time the same as the participants without PD. Other studies (Horstink et al., 1990; Gentilucci and Negrotti, 1999a) have described attentional and memory aspects that interfere with the execution of the motor plan in patients with PD and influence the information processing. Several studies have also reported sensorimotor processing abnormalities in PD (Fellows et al., 1998; Conte et al., 2017; Lee et al., 2018). The deficit in sensorimotor integration could also be observed in precision grip/lift tasks (Fellows et al., 1998). A study about arm movements that tested the effects of both visual and kinesthetic information showed that peripheral afferent feedback was impaired in patients with PD (Klockgether et al., 1995). These disturbances may also be involved in impairing the perception of distinctions between the artworks, which affected time judgment. Interestingly, it has also been suggested that basal ganglia play an important role in gating sensory inputs for guiding movements (Labyt et al., 2003).

The third aim was to investigate whether the manipulation of an artwork by PD patients and non-PD participants would present qualitative differences. Results revealed that PD patients showed the highest proportion and relative frequency of the Touch category and minor frequency of the Movement category compared to the non-PD participants. This effect may be due to the fact that PD patients present abnormalities in the execution of sequential motor tasks (Castiello et al., 1993; Gentilucci and Negrotti, 1999a,b; Alberts et al., 2000). Gentilucci and Negrotti (1999a) studied, in PD patients and healthy control subjects, the kinematics of the action formed by two successive motor acts: reaching-grasping an object and placing it on a second target. Results pointed out that PD patients were able to compute the general program of an action. However, they presented a decay of the motor program; consequently, they reprogrammed movement during its implementation. According to the authors, basal ganglia can be involved in storing the plan of an action and in controlling its correct execution. Hence, it is possible to consider that, in our study, a greater quantity of touches performed by PD patients were related to the effect of the movement reprogram, leading to difficulties in performing other movement categories. Moreover, the movements used may have influenced time perception. The task of exploring the informational content of an artwork through multiple touches is qualitatively different from exploring it through a sequence of movements. In this case, moving/manipulating the artwork can promote a better appreciation and major assimilation of its informational content when compared to manipulation through touch. In this way, the quality of the artistic appreciation presented by PD patients would have produced impairment in the processing of information content; that would have resulted in the shortening of remembered duration, which would be in agreement with the theory of storage (Ornstein, 1969).

To conclude, we observed that the PD patients showed a greater proportion of Touch for the artwork with a higher level of complexity and a minor frequency of Movement for the artwork with a lower level of complexity, whereas the non-PD participants showed the opposite. Berlyne (1958), Berlyne and Crozier (1971), and Berlyne and Lawrence (1964) stated that when the level of complexity of a stimulus is higher, it will receive more attention. The explanation is that complex stimuli attract exploratory responses and other investigative behaviors, and that would trigger an impulse, which would be promptly canceled by the inspection of the stimulus. However, Berlyne (1963) also found that the longer exposure time of pairs of images with different levels of complexity decreased the probability that the more complex figure would be chosen for a better inspection. According to Berlyne (1963), the contrast between this result and the previous results is compatible with the view that there is a preferred optimal informational content to which subjects seek to expose themselves. Considering Berlyne (1957, 1958, 1963), Berlyne and Crozier (1971), and Berlyne and Lawrence (1964), it is possible to infer that the more complex artwork exceeded the preferred optimal informational content of the non-PD participants in our study, which led them to move the less complex artwork more and to touch the more complex artwork less. On the other hand, PD patients touched the more complex artwork more than they moved it, which denotes a difference in the quality of the manipulation in comparison to non-PD participants. This may have contributed to the fact that the preferred optimal informational content was not exceeded; hence, the exploratory attention had the more complex artwork as a focus. In this way, Berlyne’s findings that complex stimuli trigger exploratory behavior, provided they do not exceed the optimal information content, were confirmed by our results.

In general, the effect of the artworks’ manipulation on the time perception demonstrated that PD patients present alterations in the verbal temporal estimation of the duration of the artworks’ manipulation with different levels of complexity. Importantly, PD patients showed a shorter duration of the artwork manipulation, and they presented qualitative differences in the manipulation of artworks as indicated by analyzing the manipulation categories. It is possible that the basal ganglia thalamocortical system is involved in motor-timing operations, which present abnormal activity in PD patients (Harrington and Jahanshahi, 2016). Concomitantly, it is possible in the present study that PD patients were more accurate in temporal estimation than healthy participants due to they received their usual medication, which included L-dopa (precursor to the neurotransmitter dopamine).

The results of this study recommend some manipulation for future studies. Comparisons analyzing the complexity of the stimulus showed moderate to large Effect size for non-statistical significance within the PD group and intergroup conditions. Besides, the Effect size was small for statistical significance of intergroup comparisons of the category of the movement Touch. The actual meaning of the data can be improved by studies that consider larger samples, data reported from other experimental studies that would be performed on similar issues, and also the context involved in subjective time, art appreciation, and Parkinson’s disease.

This study used the verbal report procedure to assess subjective time. The analysis of the questions of this research would be enriched with other procedures, such as temporal reproduction (Nather and Bueno, 2011) or temporal bisection (Nather et al., 2011). Despite the difficulties they may offer to PD patients, studies have shown that these mechanisms of time estimation are differently perceived by this population (Malapani et al., 1998; Jones et al., 2008; Harrington et al., 2011; Dušek et al., 2012; Mioni et al., 2018). The manipulation of artworks, especially those made with intent to be manipulated, is a strategy to relate subjective time, artistic appreciation and PD. This strategy can be improved through environmental and contextual manipulation, for example, listening to music during the manipulation of the artwork, different levels of familiarity with the artwork or hedonic values. So, the important role of the environment on the subjective time and behavior of PD patients can be examined with conditions of greater ecological importance.

## Ethics Statement

This study was carried out in accordance with the recommendations of The National Commission for Research Ethics (CONEP-Brazil), with written informed consent from all subjects. All subjects gave written informed consent in accordance with the Declaration of Helsinki. The protocol was approved by The Research Ethics Committee of the Faculty of Philosophy, Sciences and Letters of Ribeirão Preto, University of São Paulo, Ribeirão Preto, SP, Brazil. Patients with a diagnosis of PD were recruited from the Ribeirão Preto School of Medicine Hospital, São Paulo, Brazil. Exclusion criteria included possible dementia or cognitive impairment, significant history or current psychiatric disorders, historic of neurological disease or any condition (e.g., depression) that would interfere with testing. See Data Sheet 1 (Statement of the Commission of Ethics in Research – CAAE: 41620515.6.0000.5407) and Data Sheet 2 (Free Consent Term – Patient).

## Author Contributions

MM and JB designed the study and analyzed the data. MM and VT performed the experiments. MM wrote the article with input and reviews from JB and VT. All authors gave final approval of the version to be published.

## Conflict of Interest Statement

The authors declare that the research was conducted in the absence of any commercial or financial relationships that could be construed as a potential conflict of interest. The handling Editor declared a shared affiliation, though no other collaboration with the authors, JB and MM, at the time of review.
